# Affordable Open-Source Quartz Microbalance Platform for Measuring the Layer Thickness

**DOI:** 10.3390/s22176422

**Published:** 2022-08-25

**Authors:** Adrian Matusiak, Andrzej Marek Żak

**Affiliations:** 1 Faculty of Mechanical Engineering, Wroclaw University of Science and Technology, 50-371 Wroclaw, Poland; 2 Nanores Sp. z o. o. Sp. k., 51-317 Wroclaw, Poland

**Keywords:** FTM, QCM, film thickness monitor, evaporation, sputtering

## Abstract

The layer thickness measurement process is an indispensable companion of vacuum sputtering and evaporation. Thus, quartz crystal microbalance is a well-known and reliable method for monitoring film thickness. However, most commercial devices use very simple signal processing methods, offering only a readout of the frequency change value and an approximate sputtering rate. Here, we show our concept of instrument, to better control the process parameters and for easy replication. The project uses open-source data and its own ideas, fulfilling all the requirements of a measuring system and contributing to the open-source movement due to the added value and the replacement of obsolete technologies with contemporary ones. The device provides an easy way to expand existing sputtering machines with a proper controller based on our work. The device described in the paper can be easily used in need, being a proven project of a fast, inexpensive, and reliable thin-film thickness monitor.

## 1. Introduction

A quartz microbalance uses the direct piezoelectric effect known since 1880 [1] and describes the formation of an electric induction in a solid under the influence of stresses. In this phenomenon, the polarization of the resulting electric field depends on the type and direction of stresses, not only on the absolute value of the stress tensor. The proportion between the electric field strength and the value of the tensor is direct and linear [2]. The linearity of this phenomenon makes piezoelectric components ideal transducers and enables the use of such transducers in sensor and actuator designs. A small disc is excited by an external oscillator and resonates at a known frequency, related to its mass. During a controlled process, we place additional material on the crystal, increasing this mass. The frequency feedbacks to the control system and is processed as an analog signal, which allows us to measure the change in mass, and thus the thickness of the deposited material. A quartz microbalance is not only a sensor but also an actuator [3]—it creates a strong gravitational field around its surface. Deposited material adsorbs onto the crystal surface, changing its mass and therefore the vibration frequency. The material used in piezoelectric transducers is quartz crystals, mainly α-quartz [4], an allotropic variety characterized by a trigonal unit cell [5]. The transducer uses the linear dependence of the frequency of vibrations on the mass [6].

The quartz crystal microbalance (QCM) is a popular instrument to measure layer thickness in microscopy and thin film formation. The technology is also used to measure biofilms [7]. In addition to this, its applications include not only measurements of film thickness, but also measurements of the number of biological substances such as viruses [8]. QCM with dissipation monitoring (QCM-D) could be used not only as a biosensor but also as an immunosensor [9,10]. In addition to life science applications, the device is used to measure the properties of different layers of materials, among others, titanum carbide [11], and graphene [12], or to detect heavy metal ions [13]. Proper setups allow users to measure vapors [14,15] or measure viscosity [16] or even lubrication in nanomachines [17,18]. Measurement of water contamination [19] or nanoaerosols [20] is another example of the use of QCM in more complex sensoric systems. The technology can even be used in space. NASA prepared reports examining Mars samples [21] using this measurement method.

We discovered that many thickness monitors are closed solutions that cannot be modified or tailored to the needs. Every change in commercial equipment of sensor systems of this class requires fitting to all hardware elements, which, in this case, are mainly custom-made electronics consisting of high-sensitivity and high-quality parts. Moreover, commercial devices have strict requirements in design and cabling [22]. Adding low-quality components to the system worsens accuracy, while even top-tier electronics ensure that there is no additional impact on the measuring loop [23]. Therefore, the expected resolution might not be available. Our work finds the solution to the problem by reducing the hardware and shifting crucial elements into software implementations.

Today, measurement systems can be integrated into small devices that contain the sensor head with quartz crystal holder, the oscillator driver, and signal analysis and processing unit, usually a simple microprocessor. Due to the progressive simplification of microcontrollers, film thickness monitoring can become a relatively easy task. For this reason, we present an open-hardware and ready-to-3D-print sensor head design, as well as an electronic system with a detailed signal description necessary to build the whole QCM thickness monitor.

## 2. Materials and Methods

### 2.1. System Architecture

The creation of the system described below requires purchasing an appropriate measuring head or making it according to the model provided by the authors [24]; making a circuit board according to the electronic diagram or Gerber files created by openQCM, publicly available on their website [25]; downloading and uploading the code to the microcontroller, publicly available on the OpenQCM GitHub [26]; and downloading and running desktop application, provided by the authors [27] or another compatible software; an example can be found in the same OpenQCM GitHub.

**Figure 1:**
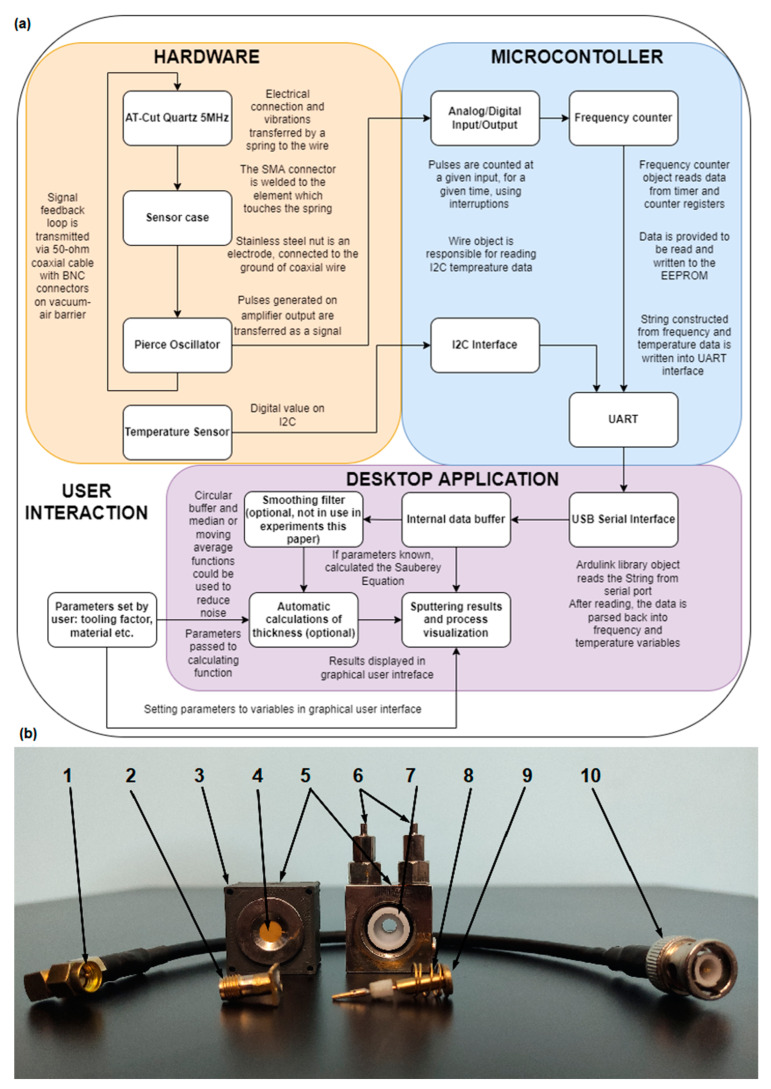
The described QCM system (**a**) hardware and logic block within the device, (**b**) physical elements of the system: 1—SMA angle plug, 2—SMA female connector with a soldered plate for fixing the connector to the case, 3—additional holes in 3D printed case for electrostatics protection, 4—quartz crystal fit inside the original metal nut, 5—hole for bayonet fitting, 6—a cooling system in an original metal case, 7—Polytetrafluoroethylene (PTFE) sleeve (in the 3D version it is a part of a model), 8—spring, 9—disk-shaped ended brass sleeve, 10—BNC connector to vacuum gland.

**Figure 2:**
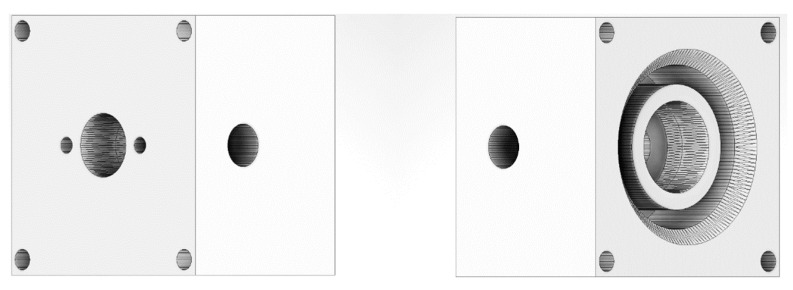
Three-dimensional model of the reversed engineered sensor head. Left—top and back view. Right—front and top view. Model available from source [24].

The described measurement systems consist of the following elements (Figure 1a):

- Sensor head, taken from another existing gauge or an open-source fused deposition modeling (FDM) 3D-printed replacement or alternative to the sensor head (Figure 2);
- Crystal oscillator circuit [25];
- Arduino Micro as a data acquisition module;
- PC or microcomputer with Universal Serial Bus (USB) interface;
- Coaxial 50-ohm cable with subminiature A (SMA) and Bayonet Neill–Concelman (BNC) connectors and USB cables and vacuum cable connector.

The sensor head is a structure consisting of a quartz holder, a brass spring, and a positioning flange inside a plastic-made dielectric sleeve, which enables the quartz crystal to resonate. When the sensor head is not dielectric, the electrode itself needs to have a galvanic connection with a transmission cable. Oscillator driver capacitors discharge all charges generated on the surface. Therefore, we recommend 3D printing accordingly to the project. The original sensor head consists of elements presented in Figure 1b: steel housing with hollow cooling channels (4), plastic sleeve (7), preload spring (8), quartz crystal (4) with an electrode (9) connected to a spring, and antenna connector, replaced with SMA type (1, 2).

The possibilities of 3D printing allow us to integrate the sleeve into one solid (Figure 2). The device is used for short-term application of thin layers, but in the case of continuous operation, it must be additionally equipped with a cooling system, especially when the 3D-printed polymer case has thermal properties worse than the original metallic one. The design shows a conventional QCM head without the possibility of cooling. Four holes were made in the corners to fit additional electrostatic protection to ensure proper discharging via the connector. Our tests provided us with data that temperature does not affect measurements on a short time scale. The main purpose of the head is to fix the crystal stably and securely.

### 2.2. Detailed Device Design

The crystals used in the device are 6 MHz AT quartz crystals 12.5 mm in diameter (Colnatec CNT05RCSG), but the design allows one to use 14 mm crystals as well—the sensor head is slightly larger than in other designs. These quartzes are popular standard replacements for film thickness monitoring systems. We recommend the use of gold coatings for the electrode to achieve better mechanical properties of the electrical connection. Other custom coatings are sometimes used in special cases, such as nanofiber coatings for safrole vapor detection [14] or many types of plastic (such as polyvinylidene fluoride) or ceramic (such as hydroxyapatite) for bioanalytics [28]. The electrical connection is crucial for the presented solution because electrical connection problems are the most common. During our tests, we encountered muffled signals. The reason was poor electrical connections caused by the scratched surface of a quartz crystal and the insufficiently stiff and precise joint between the spring and the cable connector. Due to possible problems, we recommend using standard BNC and SMA antenna connectors. The SMA connectors should be locked with screws, to prevent them from twisting. It is important because the surface of a connector is an electrode itself. Therefore, the connection between the sensor casing and the oscillator should remain intact. We recommend using coaxial 50-ohm cables. With the shorter cable, the signal is less damped.

The coaxial cable transfers the feedback signal from and to the oscillator. It is highly recommended to have a high-quality and possibly short cable. Too long a cable increases signal damping and requires additional amplifiers. Because of the need to overcome the vacuum–air barrier, the cable should be as short as possible but allow for easy positioning of the head inside the evaporator device. The wire is divided into two segments: from the crystal to the cable connector inside a vacuum, and from the cable connector to the oscillator outside the evaporator device. The connector should not have galvanic contact with the ground of the sputtering machine because it breaks the feedback loop carried by the wire. In our approach, the connector was produced on the basis of the available vacuum port of the device. Inside the drilled hole, a large diameter copper wire was insulted from the wall of the through hole with the use of epoxy resin. The large (2.5 or 4 mm) diameter requirement is because the width of the wire reduces the negative effect of the parasitic capacitance of the cable, which creates a second, parallel feedback loop and introduces additional high-frequency oscillations that we cannot measure and distort our measurement. The feedback loop from the crystal oscillator circuit is transmitted via this cable, and by meeting these requirements, the signal amplifiers are unnecessary in the system.

The signal generator is located on the printed circuit oscillator board. The feedback loop mentioned in the last paragraph connects the fields marked on the electronic diagram with X1 and X2. These fields are part of the crystal driver, the Pierce oscillator, working with quartzes up to 10 MHz. To change the range of measured frequencies, the capacitors need to be replaced. In addition, the microcontroller unit should have a proper clock, according to the Nyquist law. Our device is clocked by a 16 MHz quartz, and therefore the correct band is 8 MHz. It can be shifted from the 0–8 MHz range to 8–16 MHz using aliasing. The Y output signal of this circuit is passed on to the microcontroller, as is the temperature signal, measured with the MCP9808 sensor type thermistor [29]. The sensor communicates with the microcontroller unit (MCU) through the Inter-Integrated Circuit (I2C) interface. The output signal from the oscillator driver is connected directly to the Arduino module.

In our device, Arduino Micro is the microcontroller unit, preprogrammed to count the frequency of the signal using an inbuilt analog–digital converter (ADC). Any Atmel AVR or ARM architecture processor model can work properly, but to reduce costs and time, we recommend using Arduino or similar prototyping platforms. The module counts pulses from the resonating crystals and sends the data to a serial port along with the temperature. The frequency data transfer via the Universal Asynchronous Receiver/Transmitter (UART) interface using the USB cable to a PC or Raspberry Pi board on which the GUI application is running. This application works as a controller—we start and finish the measurement and record the data. The block diagram of the entire system is in (Figure 1a).

The software includes the Adafruit library, therefore, to operate the MCP9808 temperature sensor [30]. The library FreqCount measures the frequency of a signal with the help of interrupts [31]. The library is stable and was created by Paul Stoffregen, creator of Teensy Boards, which is used in more accurate applications. This code is also provided in the supplementary material. Transmission through the serial interface takes place at 115,200 baud. The temperature sensor object was created as a global variable when the program initializes, a single instance of the object is created, the sensor is reset, and the measurement starts. In the main program loop, the inbuilt analog–digital converter transforms information for each iteration, and the frequency data buffer to the serial interface using electrically erasable programmable read-only memory (EEPROM).

Measurement of this frequency, relative to the frequency before starting the sputtering process, with a correctly calibrated balance gives us a precise result of the average mass deposited on the crystal surface. To take the precise measurement of the sample placed inside the vacuum chamber, it is necessary to perform the appropriate calculation of the mass evaporated or sputtered onto the crystal relative to the process on the prepared sample. This factor can be called the tooling factor and is usually a percentage, and its determination is part of the calibration of the measurement system [32]. During sputtering, this parameter scales the value to estimate the thickness of the layer on the sample, based on the distance difference between the material source and the quartz crystal and between the crystal and the samples prepared.

The sequence of steps taken to make a proper measurement:

- Put the quartz crystal into a holder and read the initial stable frequency;
- The thin layer of graphite carbon evaporates onto the crystal surface;
- The stable frequency value after the process is read from the device, allowing us to calculate the frequency drop;
- Therefore, the mass and thickness of the carbon layer are calculated using the Sauerbrey equation [33].

After carbon deposition, the gold layer was sputtered in the same sequence of steps without changing the crystal. The described device can provide online measurements, thus calculating the rate of change in the sputtering process. After that, the crystal was cross-sectioned using focused ion beam scanning electron microscopy (FIB-SEM). FEI Helios Nanolab 600i with Ga source device was used to measure the real thickness of the gold layer. Independently, another experiment was performed: sputtering gold in stages with a constant rate at the QCM and geological samples. They were placed at a distance twice as far as the QCM, so the thickness of the layer on them should reach about half the thickness of the measuring device.

## 3. Results

To measure the thickness of the layer, we need to measure the initial and final frequency, which can be expressed by the formula:

(1) $$f=-\frac{2f_{0}^{2}\Delta m}{A\sqrt{\rho \mu }},$$

where *f = f*_1_ is the final frequency, *f*_0_ is the initial frequency, A is the area between electrodes, *ρ =* 2.65 g/(cm^3^) is the density of quartz, *μ =* 44 GPa is the shear modulus of quartz, and ∆*m* is mass change. Therefore, by transforming Formula (1), the mass change can be expressed by the following formula:

(2) $$\Delta m=-A\sqrt{\rho \mu }\frac{f_{0}-f_{1}}{2f_{0}^{2}},$$

Knowing the mass change, we can finally calculate the thickness of the measured layer:

(3) $$h=\frac{\Delta m}{A\rho_{m}},$$

if we know the *ρ_m_*-density of the deposited material. All uncertainties in the physical quantities described above are further discussed in the “Discussion” section.

**Figure 3:**
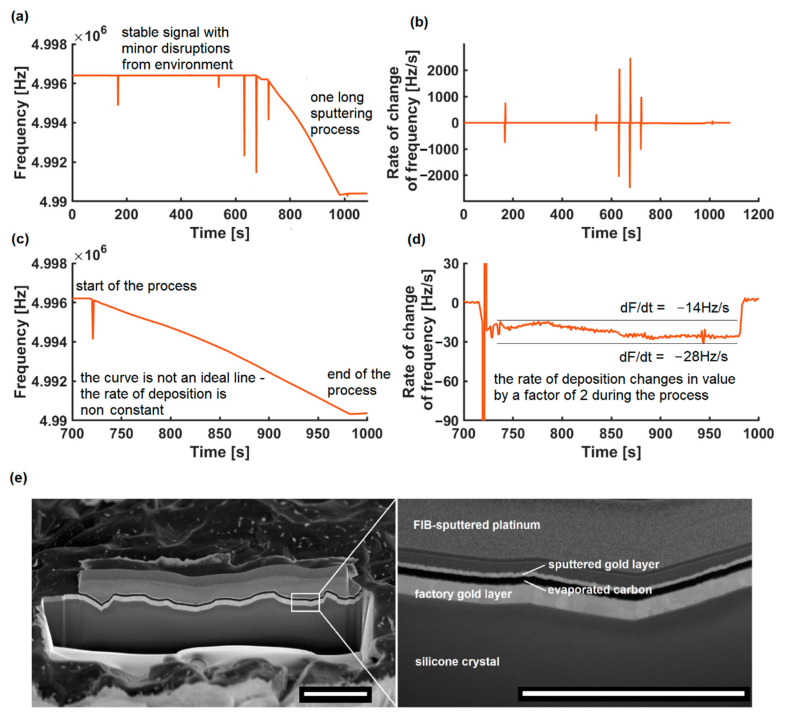
The comparison of sputtering process on QCM measurement and FIB-SEM imaging: (**a**) measured frequency; (**b**) the rate of change in the sputtering process presented on (**a**); (**c**) magnified view for time t = [700, 1000] s, showing the one deposition stage; (**d**) non-constant rate of change during deposition in the time interval; (**e**) FIB-SEM cross section of examined quartz crystal made after the experiment, scalebar 2 µm.

**Figure 4:**
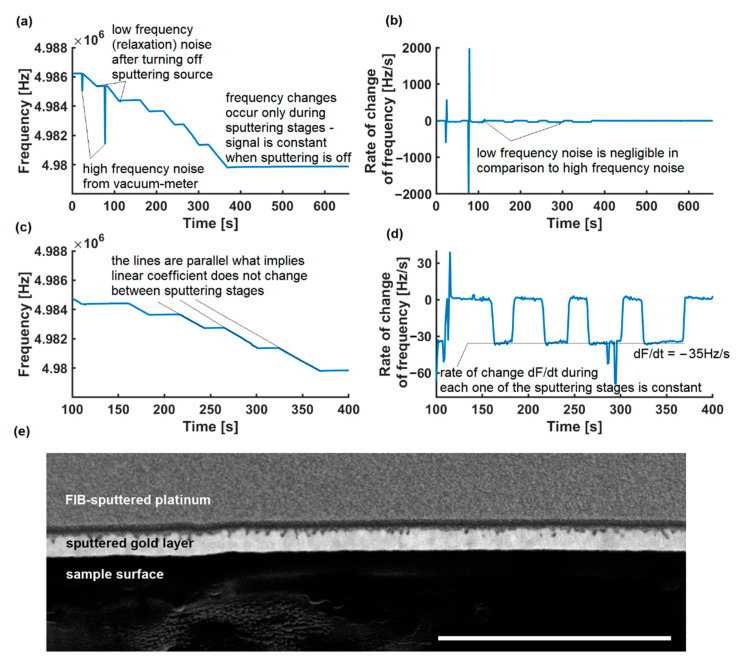
The process of gold sputtering, is divided into stages: (**a**) The frequency signal during experiment; (**b**) the rate of change during experiment; (**c**) magnified view for time t = [100, 400] s, showing the tangent lines during deposition stages are parallel; (**d**) the rate of change during deposition in the time interval is constant unlike in Figure 3; (**e**) FIB-SEM cross section of examined rock sample, scalebar 1 µm.

The starting frequency for carbon deposition was 4,997,235 Hz, and the frequency change is −827 Hz. The area between the electrodes is a circle of 6 mm in diameter. Then, the value of the thickness of the graphite layer is 79 ± 2 nm. The layer in the photo (Figure 3e) is 33 px wide, which corresponds to 74.3 nm. The standard deviation of 30 samples is 2.9 px, which corresponds to 6.5 nm. The result with extended uncertainty for a 95% confidence interval is eventually 74.3 ± 11.0 nm, so the result is valid. The experiment was performed offline; therefore, we cannot show the plot versus time. After that, we performed the gold deposition on Edwards Vacuum Coater Model 306, which we equipped with a thickness meter. Figure 4a shows the original unfiltered time series and Figure 4b shows the rate of change. The starting frequency for gold deposition is the ending frequency of carbon deposition; thus, 4,998,062 Hz. The frequency change is −6136 Hz and, therefore, the thickness of the gold layer measured by QCM is 68 ± 2 nm, while measured in the SEM image is 40.6 nm (Figure 3c). The standard deviation of 30 samples is 4.6 nm; therefore, for a 95% confidence interval, we interpret the result as 40.6 ± 9.2 nm. This is a significant discrepancy from the theoretical assumptions. However, as shown in Figure 3a, the instrument is very sensitive when the Penning-type vacuum gauge is turned off and on. It is powered by high voltage, and its appearance or disappearance induces a significant alternating magnetic field, temporarily affecting the meter reading. To ensure a reliable reading under in situ conditions, it is known practice to obscure the formulation prior to establishing stable coating conditions and, after stabilizing the thickness gauge, resetting it to zero while exposing the actual formulation. Then, while maintaining the same evaporation rate, it is possible to more accurately estimate the thickness of the layer.

The process with a more constant rate of change is presented in Figure 4, and is, moreover, an example of the real working conditions of the device. For this purpose, we placed the QCM at a distance of approximately 50.7% (no known uncertainty of the distance) of the target-sample distance, and the sputtering object was a geological cross section. The read value of the initial frequency was 4,998,005 Hz, the read value of the final frequency was 4,981,705 Hz; therefore, the gold layer on the crystal is 251 ± 2 nm. The value of the gold layer on the rock sample is 127 ± 14 nm and, taking into account the correction factor related to a different distance from the target (1/50% = 200%), the estimated value in the sample is 254 ± 29 nm. The calculated value of uncertainty is not A (type A uncertainty—1.2% in the experiment), but rather type B uncertainty. Type A uncertainties will be described, but will not be discussed here as it is not a feature of our system, rather an experimental random error. If we ensure the exact target-sample distance, the problem will be eliminated.

## 4. Discussion

At this point, we would like to describe further the measurement uncertainties that have accompanied our discussions so far. To verify the work of QCM, we performed a physical measurement of the thickness of the sputtered layers using the FIB-SEM method. The results obtained correspond to cross sections; although, there are some inaccuracies to discuss. The problem with properly verifying this measurement is the electron- and ion-deposited platinum layer, which is necessary to provide a cross-sectional cut. The platinum used in the process forms a solid solution with gold, which can locally change the measured value. It is worth noting that the measurement value for the much lighter and more QCM-demanding carbon remained correct. However, the probable differences are not significant and this affordable system can be useful in estimating the value of various processes. The effect of ion-platinum alloying needs further examination or using an additional carbon layer before the FIB-SEM measure. The formulas used to calculate the thickness and uncertainty were constructed from the Formulas (1)–(3), written in the previous chapter.

First, to discuss these results, we need to estimate the sensor area, part of the crystal surface that is not covered by the uncertainty (symbol—[A] in Formulas (1)–(4)) of the crystal. The uncertainty of the area is based on taking measurements using a laboratory caliper (Δx=0.02 mm). Because all the values of a nut hole diameter obtained were 6.00 mm, we decided to go for type B uncertainty. This is example 1 of an equation:

(4) $$u\left(d\right)=\sqrt{\frac{{\left(\Delta x\right)}^{2}}{3}}=\sqrt{\frac{{\left(0.02\mathrm{mm}\right)}^{2}}{3}}=11.55\;\mu m,$$

Therefore:

(5) $$u\left(A\right)=\sqrt{{\left(\frac{\partial \left(\frac{\pi }{4}d^{2}\right)}{\partial d}\right)}^{2}\times u{\left(d\right)}^{2}}=0.11\;{\mathrm{mm}}^{2}$$

The same principle applies to estimating the frequency uncertainty. We encounter noise during experiments, so we add them as statistical noise. For a few thousand samples we use Gaussian distribution, not the t-Student one. Therefore, the expanded uncertainty is obtained by multiplying by the factor of *k* = 2. The resolution of the device Δ*f* is 1 Hz. The standard deviation of the noise is 1.82 Hz. We see here that type A is a larger source of uncertainty than type B. However, there are still many possibilities to increase accuracy by reducing noise. The type B uncertainty of frequency measurement is given by a formula:

(6) $$U_{b}\left(f\right)=\sqrt{\frac{{\left(\Delta f\right)}^{2}}{3}}=0.59\;\mathrm{Hz},$$

The expanded type A is calculated using multiplying factor *k*:

(7) $$U_{s}\left(f\right)=k*s=3.64\;\mathrm{Hz},\;$$

Therefore, the calculated uncertainty is eventually equal to:

(8) $$u\left(f\right)=\sqrt{U_{b}{}^{2}+U_{s}{}^{2}}=3.68\;\mathrm{Hz}$$

The first term in the sum under the radical is 3.87 × 10^−22^ kg^2^, the second is 1.39 × 10^−29^ kg^2^, and the third is 1.26 × 10^−22^ kg^2^. The conclusion is that the uncertainty of the measurement of the initial frequency is negligible. The main sources are geometry and the final frequency (frequency change). The mass uncertainty calculated in this way is 2.26 × 10^−11^ kg. The value is almost 200 times lower than the original OpenQCM project mass sensitivity. The main inaccuracy comes from both mass measurements and geometrical dimensions of the crystal (area). To sum it all up:

(9) $$u\left(h\right)=\sqrt{{\left(\frac{\partial h\left(\Delta m,\;A\right)}{\partial \Delta m}\right)}^{2}*\;u{\left(\Delta m\right)}^{2}+{\left(\frac{\partial h\left(\Delta m,\;A\right)}{\partial A}\right)}^{2}*\;u{\left(A\right)}^{2}},$$

(10) $$u\left(h\right)=\sqrt{{\left(\frac{1}{A\rho_{m}}\right)}^{2}*\;u{\left(\Delta m\right)}^{2}+{\left(\frac{\Delta m}{A^{2}\rho_{m}}\right)}^{2}*\;u{\left(A\right)}^{2}}$$

The first term under the radical, uncertainty of mass, squared is equal to 5.50 × 10^−19^ m^2^, and the second term, uncertainty of electrode area, is 4.15 × 10^−19^ m^2^, which implies that elements should be taken into an estimation of the result. The final result is 0.982 nm, which is approximately 1 nm. Therefore, the maximum uncertainty type B from the device is under 1 nm, and extended uncertainty is 2 nm. Knowing this, we can say that, using our device, all errors will come from the operator, method, and statistics, not the device itself.

The measurement is thermally stable. Temperature does not affect accuracy in a short time, and the characteristic function describing the transducer is almost ideally linear in the thickness range tested. The temperature of electronics is stable; therefore, there is no need to compensate for the temperature drift of electronic components. Although the device can count every pulse, and the type B uncertainty of the device is equal to high-tech industry standards, which is about a 1 nm accuracy, the final inaccuracy is much larger and comes from the following aspects.

The first is lacking knowledge of the exact properties of the material—for example, the density of graphite is in the range of 2.09 to 2.23 g/cm^3^, with an average of 2.16 g/cm^3^ [34]. Some sources describe the specimens with even higher density. The density theoretically depends on the temperature, which was 29 °C in our test, while most of the benchmark experiments try to achieve 20 °C, but in this case the impact of the temperature on the material is negligible. Polycrystalline graphite subjected to pyrolysis has a density of 2.2 g/cm^3^ [35]. The article is based on the average value of 2.16 g/cm^3^. The type B uncertainty of the measurement is 0.936 nm for the lower boundary value of the graphite density of 2.09 g/cm^3^ and 1.015 nm for 2.23 g/cm^3^. It decreases with the density, so the device should be more accurate for more dense gold. Assuming the pessimistic cases for uncertainty sources, 1 nm is an unextended uncertainty type B, and 2 nm is an extended one.

During the process, as a result of the sputtered layer, the density and area of the electrode are constantly changing. It is important, especially for high-density materials. The proposed solution is to calculate the mass in each step recursively, therefore changing the density value in internal memory, using a weighted average. Furthermore, as we see in the microscope picture, the golden electrode of the quartz crystal affects the mean density of the sensor, which is no longer 2.65 g/cm^3^. So, another uncertainty appears here. Changes are not big to scale, because the gold layer is in micrograms, but that impacts the result. The longer the crystal is used, the more depositions are made, and the difference increases. The best idea to address this problem is to verify the average density of the whole quartz crystal, before the first measurement, and store the adjusted density value after the last measurement when the device is turned off. After several dozen sputtering processes, the quartz should be replaced, because it would no longer oscillate properly; the damping factor comes into play with increased mass and lower signal level. The solution to this problem is the monitoring of dissipation. The measurement of the dissipation factor makes QCM an innovative biosensor and allows for such tasks as measuring flows. Therefore, it can measure lubrication in nanomachines and allows them to achieve better performance [17,18]. Knowing the exponential decay factor, we can measure the viscosity parameters [26]. Other ideas that are based on this phenomenon are biosensors for water contamination [20]. Every higher-class quartz crystal microbalance has this function implemented.

Another topic is the low sampling frequency and high acquisition time in a basic model of work: the frequency measurement comes from obtaining the number of samples in a 1-s interval. Therefore, the system is not real-time, and it is one of the reasons why some algorithms cannot overcome the problems of the QCM. Therefore, we are limited with the accuracy and the system is prone to various interruptions. The shorter the time of acquisition of frequency data, the greater the amount of noise in the frequency. The noise value down to 0.1 Hz, according to the OpenQCM website, is false and probably impossible to achieve using the standard configuration and the software provided, where the number of counted pulses is simply integers. Even with our assumption and a monitored noise of almost 4 Hz, the uncertainty remains small.

During the gold sputtering experiment, the typical stable rate of change was 35 Hz (Figure 4d) per second and due to the sampling frequency, such data could be lost. The sampling frequency could be increased when using a high-performance microcontroller unit, but it would collide with the intention of creating an affordable system. Creating a custom board with a high-performance external ADC with control registers that can send more buffered data via the I2C interface to the MCU could be an improvement here.

However, the system still gives better results than commercial systems in a range of around EUR 5000, while can be built in part of that price from easily accessible elements. The great result is that there is such a resolution with little data (one sample per second). It is a compromise between accuracy and resolution because one unit cannot simultaneously count pulses and register data. The reason is that the library uses interrupts to properly measure frequency. Using two MCU units or direct memory access (DMA) could also be an inexpensive improvement. However, with the growth of additional electronics, the system is no longer simple and easy to install. At this moment, the replication of our system is a cheaper and faster way. It is though more friendly to students and doctors because it is built of simple elements they know.

The overall costs of all elements needed to build a full measurement system include less than EUR 200 for electronics (microcontroller, driver, and cables) when assumed connection to a laptop PC. An additional Raspberry Pi microcomputer configured with a touch panel may replace a desktop computer for about EUR 200, making the solution a standalone system. Three-dimensional printing of the sensor head and casings should cost less than EUR 150. The sensor head requires metal inserts, but they could be made using hand tools; CNC machining is not required. The full project should close around EUR 600. Meanwhile, the commercial instruments with such a range start their pricing at around EUR 5000. Devices with a range starting at 100 nm can cost around EUR 1000 but do not offer modularity and expansion possibilities.

The system is highly modular. Each module can be replaced with another solution, expanded, or tailored to special needs. It is possible to use industrial standard elements with these described in the article. Any sensor head with a frequency signal output can work within the system as long as the oscillator circuit can work with this signal. The sensor head with USB signal output can work with the provided software if it is connected directly and the signal frames are valid. The oscillator circuit can also be replaced to change the frequency range—only Pierce’s oscillator work principle must remain. The same goes for microcontroller units and interfaces: it is possible to transfer data via Wi-Fi or Bluetooth, by changing the microcontroller unit and making small program changes.

**Figure 5:**
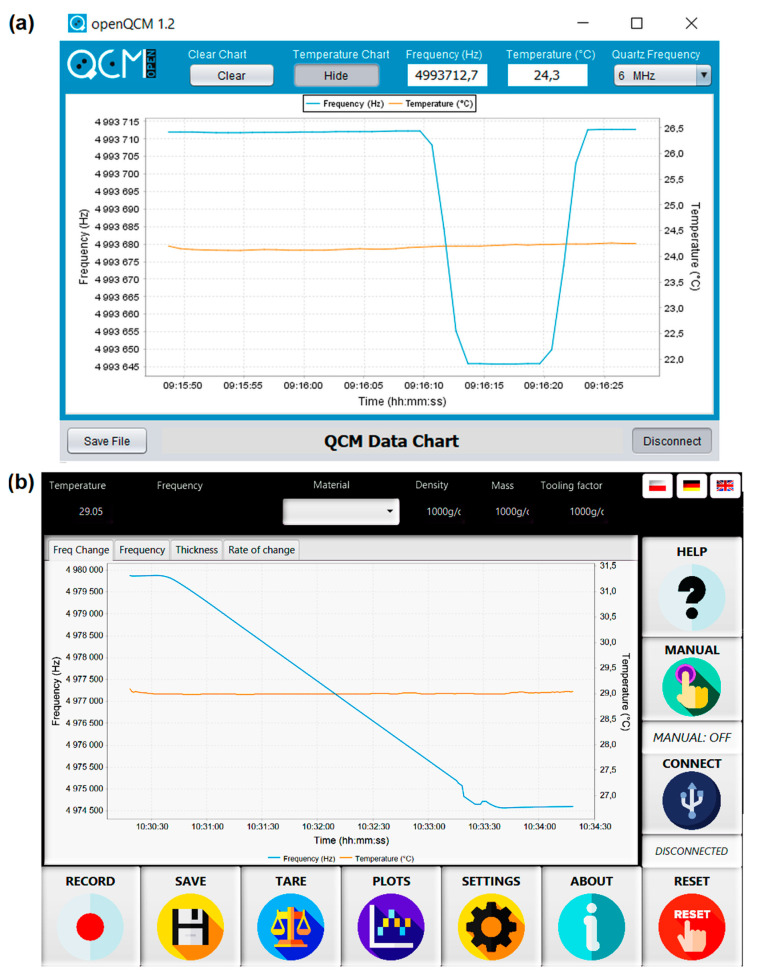
(**a**) OpenQCM application interface, (**b**) interface created for the purpose of this work, based on the same classes as interface (**a**).

The software can also be replaced with another third-party or open-source solution that can read from the interface. Our system worked well with Arduino with IDE Plots, OpenQCM software (Figure 5a), and our software (Figure 5b) as well. Each of them can be used on various systems and architectures, including all x86 and all ARM systems supporting Java (e.g., Raspbian). It does not require LabVIEW or expensive similar software, such as the commercial Prevac solutions [36]. The use of dedicated software running on an external microcomputer makes the described QCM a compact device that can store the characteristics of various materials and support the vaporization of multilayer coatings. It also allows for a live preview of the operating parameters of the resonating crystal, allowing for the identification of external measurement disturbances (Figure 3a and Figure 4a)

The great advantage of the system is that it does not require calibration. The only thing needed for the proper measurement is stable initial frequency—the measurement is relative to it, and the steepness of the curve does not depend on conditions. Small noises are acceptable and can be resolved owing to the median filter included in the software. However, during laboratory tests, this was not necessary.

Higher-class devices have systems that monitor and compensate for disturbances, mainly caused by temperature and attenuation of the resonant system itself, which are described in mathematical models, usually with a damped harmonic oscillator. There is no need to compensate for this damping, because the attenuation increases only with a large mass.

## 5. Conclusions

The differences, including the price differences, between the commercial film thickness monitor (FTM) devices are mainly the number of channels supported. However, the reliability of the OpenQCM platform allows for easy expansion to the user’s needs. The device’s goal to provide cheap and reliable measurements for sample preparation and microscope experts could be suitable even for the high-throughput control of industrial sputtering processes. The method of measuring precise layer thickness is particularly important, among others, when it comes to health and safety [8,13,14,15,20]. This paper shows how a simple QCM controller could be upgraded and well suited also to maximize the quality of measurements and user experience in typical deposition processes such as vacuum sputtering or thermal evaporation.

## Data Availability

Data will be made available on request.
